# PERSONALITY FACTORS AND ELEVATED DEPRESSIVE SYMPTOMS AMONG MEXICAN ADULTS AGED 50 AND OLDER WITH PAIN

**DOI:** 10.1093/geroni/igad104.2717

**Published:** 2023-12-21

**Authors:** Sarah Navid, Sirena Gutierrez, Joseph Saenz, Michael Antonietti, Sadaf Milani

**Affiliations:** University of Texas Medical Branch, Galveston, Texas, United States; University of California San Francisco, San Francisco, California, United States; Arizona State University, Phoenix, Arizona, United States; University of Miami, Miami, Florida, United States; University of Texas Medical Branch, Galveston, Texas, United States

## Abstract

Older adults with pain are at risk for developing elevated depressive symptoms. Personality factors, including internal locus of control (LOC) and conscientiousness, have been associated with fewer depressive symptoms in older adults. Our objective was to examine LOC and conscientiousness as potential buffers of depressive symptoms among Mexican adults aged 50 and older with pain. We used data from the Mexican Health and Aging Study (2015-2018). We included participants who reported frequent pain at both waves (n=2,211). LOC and conscientiousness were measured continuously, with higher values indicating a more internal LOC or conscientious personality. We incorporated depressive symptoms from both waves to create a 4-level depressive symptoms variable: stable low/ no depression (< 5/< 5), recently remitted (5+/< 5), recent onset (< 5/5+), and stable high (5+/5+). We used multinomial logistic regression models to assess the association between personality and depressive symptoms, adjusting for sociodemographic variables. At baseline, participants (73.2% women) were on average 67.6 years old. A more internal LOC was associated with a lower risk of stable high depressive symptoms compared to those with stable low/no depression symptoms [Relative Risk Ratio: 0.82, 95% confidence interval: 0.72, 0.92). Both personality factors were not associated with recently remitted or recent onset depressive symptoms. In a sample of older adults with pain, those with a more internal LOC had decreased risk of experiencing stable high depressive symptoms. Internal LOC may alter the perception of pain. Interventions on orienting individuals to a higher internal LOC may be helpful in pain management by mitigating comorbid depressive symptoms.

